# Unraveling the global landscape of *Elizabethkingia* antibiotic resistance: A systematic review and meta-analysis

**DOI:** 10.1371/journal.pone.0323313

**Published:** 2025-05-30

**Authors:** Saade Abdalkareem Jasim, Jaafaru Sani Mohammed, Rangaswamy Roopashree, Mohammad Y. Alshahrani, Aanchal Sharma, Abhishek Sharma, Shodiyev Asliddin, Masoumeh Beig

**Affiliations:** 1 Medical Laboratory Techniques Department, College of Health and Medical Technology, University of Al-maarif, Anbar, Iraq; 2 Medical Analysis Department, Tishk International University, Erbil, Iraq; 3 Department of Chemistry and Biochemistry, School of Sciences, JAIN (Deemed to be University), Bangalore, Karnataka, India; 4 Central Labs, King Khalid University, AlQura’a, Abha, Saudi Arabia; 5 Department of Clinical Laboratory Sciences, College of Applied Medical Sciences, King Khalid University, Abha, Saudi Arabia; 6 Chandigarh Pharmacy College, Chandigarh Group of Colleges, Jhanjeri, Mohali, Punjab, India; 7 Department of Medicine, National Institute of Medical Sciences, NIMS University Rajasthan, Jaipur, India; 8 Agronomy Navoi State University of Mining and Technologies, Navoiy, Uzbekistan; 9 Department of Bacteriology, Pasteur Institute of Iran, Tehran, Iran; Shiraz University of Medical Sciences, IRAN, ISLAMIC REPUBLIC OF

## Abstract

**Background:**

The emergence of antibiotic-resistant *Elizabethkingia* strains poses a significant challenge in clinical settings. This systematic review and meta-analysis provide a comprehensive assessment of the prevalence of antibiotic resistance among *Elizabethkingia* isolates across different regions worldwide.

**Methods:**

A systematic literature search was conducted across PubMed, Embase, Web of Science, and Scopus from 1998 to January 9, 2024, using predefined search strategies. Eligible studies reporting antibiotic resistance in *Elizabethkingia* were included. A random-effects model was applied to estimate resistance proportions and assess heterogeneity. Additional analyses, including meta-regression, subgroup evaluations, and assessments of outliers and influential studies, were performed to explore resistance trends and evaluate publication bias. Study quality was assessed using the Joanna Briggs Institute Checklist, and all statistical analyses were conducted using R with the metafor package.

**Results:**

A total of 1,016 articles were identified, of which 34 studies (47 reports) met the inclusion criteria. The pooled analysis indicated high resistance to ceftazidime (88.5% of isolates, 95% CI: 21.1%–99.5%) with no significant heterogeneity. Resistance to rifampin was 12.5% (95% CI: 5.9%–24.7%), showing substantial heterogeneity, while ciprofloxacin resistance was 27.9% (95% CI: 13.8%–48.4%) with considerable heterogeneity. Among specific antibiotics, cefotaxime resistance was 96.1% (220 isolates), meropenem resistance was 92.4% (353 isolates), and gentamicin resistance was 77.7% (356 isolates). Additionally, sulfamethoxazole resistance was 46.1% (176 out of 360 isolates, 95% CI: 23.5%–70.4%), displaying significant heterogeneity.

**Conclusion:**

This study highlights the widespread antibiotic resistance in *Elizabethkingia*, underscoring the urgent need for targeted treatment strategies and enhanced surveillance programs. The findings emphasize the importance of monitoring local resistance patterns to guide clinical decision-making. Future research should focus on elucidating resistance mechanisms and genetic diversity to develop practical therapeutic approaches and mitigate the global health impact of *Elizabethkingia* infections.

## 1. Introduction

The emergence of multidrug-resistant (MDR) *Elizabethkingia* species presents a significant global health concern, particularly in immunocompromised patients [1]. *Elizabethkingia*, a Gram-negative bacterium, is known to cause severe infections such as pneumonia, bacteremia, neonatal meningitis, and nosocomial outbreaks [2,3]. Since its discovery in 1959 by Elizabeth O. King, the genus has undergone multiple taxonomic revisions, with recent advancements in whole-genome sequencing refining its classification into eight species [4].

Antibiotic resistance in *Elizabethkingia* species, especially *E. anophelis* and *E. meningoseptica*, presents significant clinical challenges due to various resistance mechanisms [5]. These include the production of beta-lactamases, which degrade beta-lactam antibiotics like carbapenems and cephalosporins, and efflux pumps, such as the AcrAB system, to expel antibiotics like fluoroquinolones [6]. The genetic diversity among *Elizabethkingia* strains further complicates treatment, as different strains exhibit varying levels of susceptibility [7]. Additionally, the species’ ability to adapt to environmental reservoirs and acquire resistance genes from other microorganisms enhances their survival in clinical settings, particularly among immunocompromised patients. Together, these factors make managing infections caused by *Elizabethkingia* highly challenging [8]. Genetic diversity among strains further complicates treatment, with variations in susceptibility patterns and the acquisition of resistance genes enhancing their persistence in clinical settings [7,8]. Despite standardized Minimum Inhibitory Concentration (MIC) breakpoints, resistance is commonly assessed using Clinical and Laboratory Standards Institute (CLSI) M100 guidelines and European Committee on Antimicrobial Susceptibility Testing (EUCAST) PK–PD non-species breakpoints [9,10].

Recent outbreaks, such as the 2015–2016 Wisconsin cluster, have underscored *Elizabethkingia*’s adaptability and virulence, with Rapid genomic changes contributing to resistance and pathogenicity [11–13]. Intrinsic resistance mechanisms, including beta-lactamases and topoisomer modifications, further complicate antimicrobial therapy, highlighting the need for enhanced surveillance and antimicrobial stewardship [14–17]. The economic and clinical burden of *Elizabethkingia* infections, particularly in hospital settings, necessitates improved diagnostic strategies and infection control measures [18–21].

This systematic review and meta-analysis aim to comprehensively assess global *Elizabethkingia* resistance patterns, identify trends in antimicrobial susceptibility, and explore regional variations. By including studies from 1998 to 2023, we ensure a broad temporal analysis of resistance trends. Additionally, we assess the impact of testing methodologies on reported resistance rates, aiming to bridge knowledge gaps and support informed clinical decision-making in managing *Elizabethkingia* infections.

## 2. Methods

This systematic review and meta-analysis were conducted following the Preferred Reporting Items for Systematic Reviews and Meta-Analyses (PRISMA) [22] guidelines and were registered with PROSPERO (ID: CRD42025645625, https://www.crd.york.ac.uk/PROSPERO/view/CRD42025645625).

The study aimed to evaluate antibiotic resistance patterns in *Elizabethkingia* species across various geographic regions.

### 2.1. Eligibility criteria

#### 2.1.1. Inclusion criteria.

**Study design and scope:** Eligible studies included those that specifically examined *resistance patterns in Elizabethkingia*. This encompassed various study designs, such as observational studies (both prospective and retrospective), experimental research, and comprehensive surveillance reports, all of which supplied empirical data on resistance rates. Outcome Measures: The main criterion for inclusion was reporting the proportion of *resistance to Elizabethkingia*. Studies were expected to provide quantitative data on resistance rates, ideally defined according to recognized standards such as the CLSI and EUCAST guidelines.

**Sample size and population:** A minimum sample size is required for inclusion. Studies that examined *Elizabethkingia* isolates from any clinical or veterinary source were eligible, reflecting the pathogen’s relevance across different environments.

**Publication date:** The studies’ publication dates were not restricted to ensure a comprehensive analysis, allowing for the inclusion of historical data alongside contemporary findings.

#### 2.1.2. Exclusion criteria.

**Study design limitations:** Single-arm studies were excluded because they do not provide complete data, essential for a nuanced understanding of resistance patterns.

**Incomplete data:** To maintain the integrity and reliability of the meta-analysis, studies needing more specific data on the proportion of *Elizabethkingia* resistance or those with unclear methodologies were excluded.

**Review articles and editorials:** To focus on original research data, narrative reviews, systematic reviews, meta-analyses, editorials, and opinion pieces were excluded. However, relevant insights from these sources were considered for qualitative contextualization.

**Case reports and case series:** Given their limited scope and potential for selection bias, the quantitative meta-analysis did not include case reports and small case series. However, they were noted for qualitative insights where applicable.

**Duplicate studies:** To avoid data redundancy and potential bias, studies reporting duplicated or subsets of data already covered in other publications were excluded.

### 2.2. Information sources and search strategy

From 1998 to January 9, 2024, a systematic literature search was conducted in Scopus, PubMed, Web of Science, and EMBASE. The search strategy included keywords such as *Elizabethkingia*

### 2.3. Study selection and data extraction

Duplicate records were removed using EndNote (version 21). Two independent reviewers screened titles, abstracts, and full texts based on predefined inclusion criteria. A third reviewer resolved discrepancies. Extracted data included study characteristics (authors, publication year, country, sample size), antibiotic susceptibility testing methods, and resistance rates.

### 2.4. Antibiotic breakpoints and cut-off values

This meta-analysis classified *Elizabethkingia* strains as resistant or sensitive based on the MIC values and disk diffusion (DD) zone diameters reported in the included studies. Since no officially defined breakpoints for *Elizabethkingia* exist in CLSI or EUCAST guidelines, the breakpoints were derived from commonly accepted guidelines for similar Gram-negative bacteria such as *Pseudomonas aeruginosa*, *Enterobacteriaceae*, and other related pathogens.

### 2.5. Risk of bias assessment

Study quality was assessed using the Joanna Briggs Institute (JBI) Checklist [23]. Two independent reviewers evaluated study reliability, and disagreements were resolved through discussion.

### 2.6. Statistical analysis

To estimate the pooled prevalence of antibiotic resistance, a random-effects model was applied, incorporating both within-study and between-study variability. Statistical analyses were performed using R (version 4.2.1) and the metafor package (version 3.8.1) [24–31]. Heterogeneity across studies was assessed using Cochran’s Q-test and the I² statistic, where a significant Q-test (p < 0.05) indicated heterogeneity, and I² quantified the proportion of total variation due to differences between studies (P2). Statistical significance was determined using different p-values for various comparisons: P1 represents the p-value for the difference from a zero-resistance rate, P2 denotes the p-value for heterogeneity between reports. Subgroup analyses explored variations in resistance proportions across geographic regions, study years, species, and susceptibility testing methods, with p-values for these comparisons reported as P3. Meta-regression analysis was conducted to examine temporal trends in resistance rates. Publication bias was assessed using rank correlation (Begg’s test) and regression analysis (Egger’s test), with funnel plot asymmetry examined to detect bias. The fill-and-trim method was applied to adjust for potential missing studies. Sensitivity analyses were performed by identifying and excluding influential studies through Studentized residuals and Cook’s distance to ensure the stability of pooled estimates.

### 2.7. Assessment of reporting bias and certainty

To evaluate potential reporting bias, we employed rank correlation and Egger’s regression tests to detect funnel plot asymmetry. Additionally, we applied the Fail-Safe N and Trim-and-Fill methods to mitigate the impact of publication bias and enhance the robustness of our findings. These complementary approaches further strengthened the reliability and credibility of our conclusions.

## 3. Results

### 3.1. Study selection

**Fig 1:**
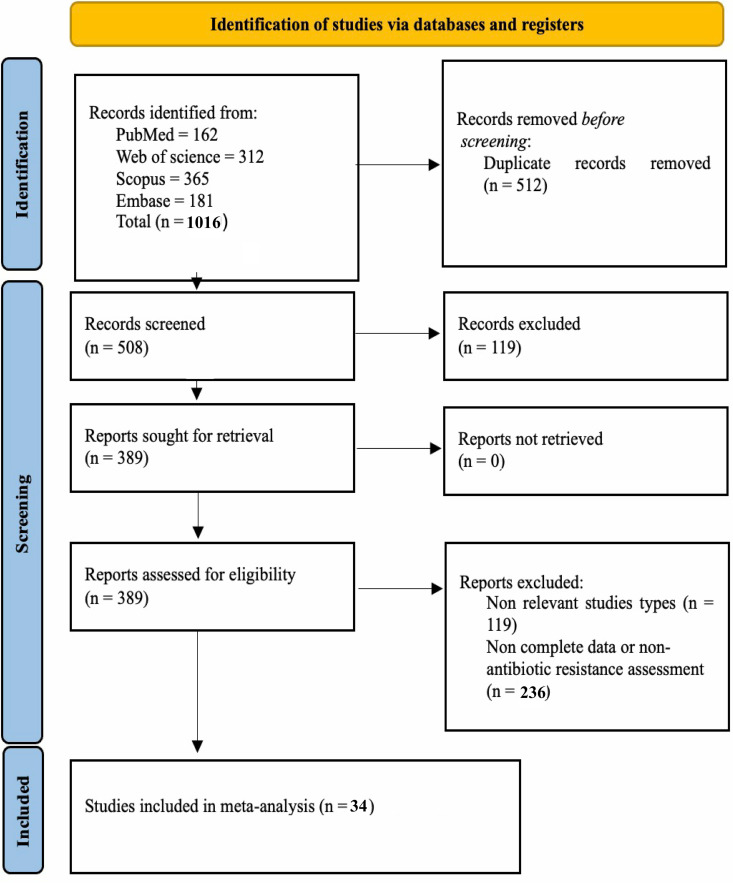
The PRISMA flowchart provides an overview of the article selection process, detailing the steps of identification, screening, eligibility assessment, and final inclusion of studies.

A total of 1,016 studies were retrieved from Scopus, PubMed, EMBASE, and Web of Science. After removing 512 duplicates, 119 non-relevant articles and non-English publications were excluded. Following the full-text screening, 34 eligible studies were included in the final analysis [32–65]. PRISMA flowchart illustrates the selection process (Fig 1 and S1 Table in S1 File).

### 3.2. Study characteristics

Between 1998 and 2023, the included studies were conducted in seven countries, including Asia, Africa, Europe, North America, South America, and Oceania, covering all four continents and studies spanning multiple continents. The studies employed a variety of antimicrobial susceptibility testing (AST) methods and followed different guidelines. Prevalence of Antibiotics resistance

### 3.3. Prevalence of antibiotic resistance

The pooled antibiotic resistance rates were estimated using a random-effects model, with heterogeneity assessed through I² and Q-tests.

#### 3.3.1. Prevalence of ceftazidime resistance.

**Fig 2:**
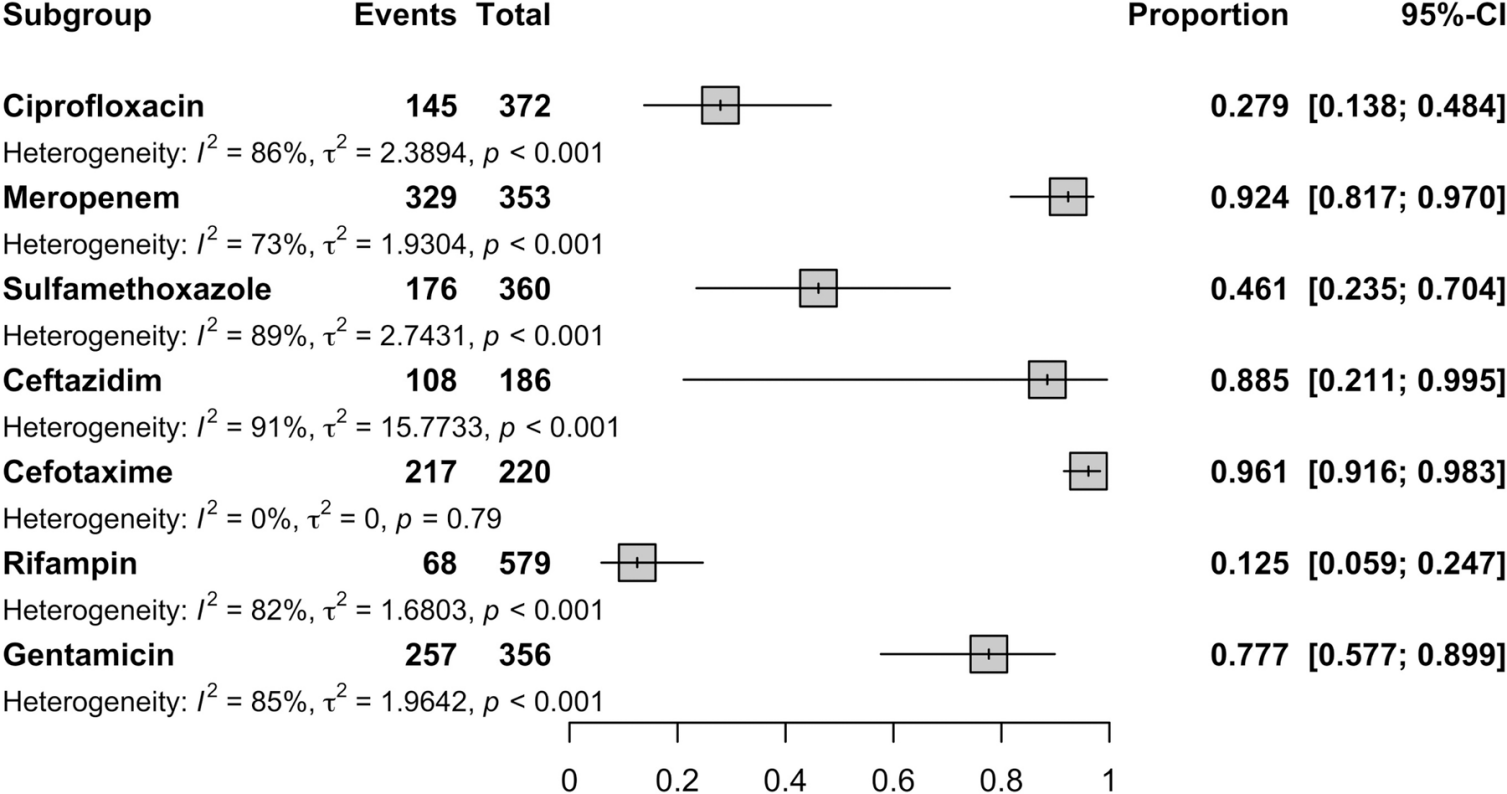
A forest plot depicting the overall proportion of antibiotic-resistant *Elizabethkingia* isolates (Ceftazidime, Rifampin, Ciprofloxacin, Cefotaxime, Meropenem, Gentamicin, and Sulfamethoxazole), calculated using a random-effects model.

**Fig 3:**
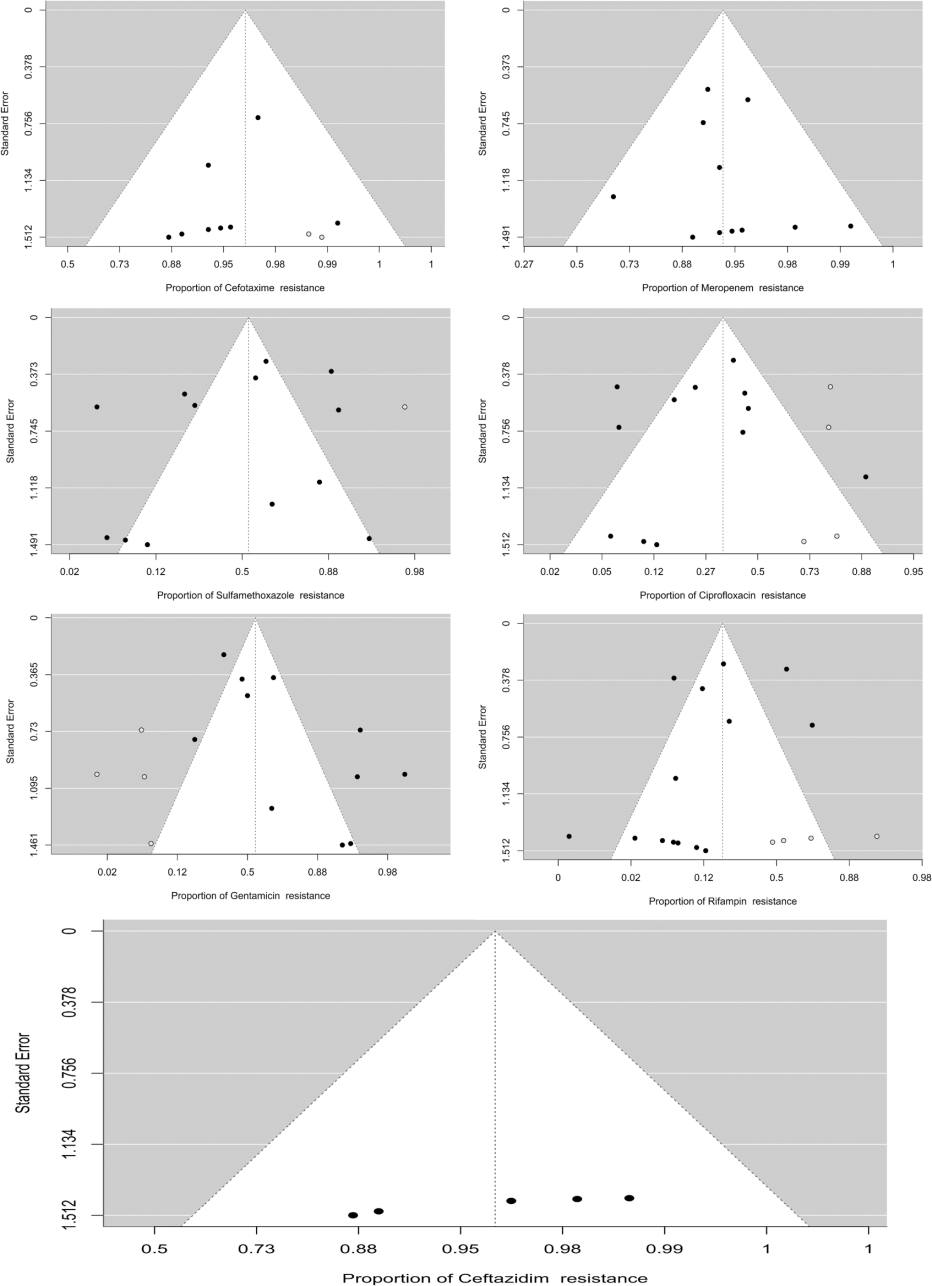
Funnel plots for assessing publication bias in antibiotic resistance studies. These plots visualize the relationship between study precision and effect estimates for various antibiotics. The symmetrical distribution around the central vertical line indicates minimal publication bias. To address potential asymmetry, the trim-and-fill method was applied. This method involves imputing studies for symmetry, represented by white circles. The adjusted resistance proportions post-Trim and Fill are as follows: Ceftazidime (0.703, 95%CI 0.144-0.971), Rifampin (0.185, 95%CI 0.095-0.330), Ciprofloxacin (0.372, 95%CI 0.203-0.580), Cefotaxime (0.968, 95%CI 0.934-0.985), Meropenem (0.880, 95%CI 0.753-0.946), and Gentamicin (0.557, 95%CI 0.327-0.764).

A total of 186 isolates from six studies were analyzed. The estimated resistance proportion was 0.885 (95% CI, 0.211–0.995), with significant heterogeneity (I² = 90.91%, p < 0.001). The fill-and-trim method adjusted the estimate to 0.703 (95% CI, 0.144–0.971). One potential outlier was identified [66], and exclusion resulted in a revised estimate of 0.703 (95% CI, 0.144–0.971) (Table 1). A forest plot showing the observed outcomes and the estimate based on the random effects model is shown in Fig 2. Funnel plot asymmetry was suggested by the rank correlation test (p = 0.017) but not by the regression test (p = 0.117) (Fig 3).

**Table 1:** Evaluation of publication bias in meta-analysis.

Antibiotic	Egger Test	Begg test	Fail and safe	Trim and Fill
Ceftazidime	p < 0.001	p = 0.469	9	0.703 (0.144, 0.971)
Rifampin	p = 0.046	p = 0.552	490	0.185 (0.095, 0.330)
Ciprofloxacin	p = 0.193	p = 0.928	114	0.372 (0.203, 0.580)
Cefotaxime	p = 0.504	p = 0.031	144	0.968 (0.934, 0.985)
Meropenem	p = 0.140	p = 0.459	340	0.880 (0.753, 0.946)
Gentamicin	p = 0.002	p = 0.191	72	0.557 (0.327, 0.764)
Sulfamethoxazole	p = 0.428	p = 0.765	0	0.537 (0.286, 0.771)

This table provides a comprehensive assessment of potential publication bias in the meta-analysis using a range of statistical techniques. Included are statistics generated from Egger’s Method, Begg’s Method, the Fail-Safe N (NFS), and the Trim-and-Fill Method. These methods are applied to investigate the presence of bias and its impact on the meta-analysis results, ensuring the robustness and reliability of the findings.

#### 3.3.2. Prevalence of cefotaxime resistance.

Among 220 isolates across eight studies, the estimated resistance proportion was 0.961 (95% CI, 0.916–0.983), with no significant heterogeneity (I² = 0.00%, p = 0.789). The fill-and-trim method adjusted the estimate slightly to 0.968 (95% CI, 0.934–0.985). No outliers or overly influential studies were detected (Cook’s distance). Funnel plot asymmetry was indicated by the rank correlation test (p = 0.031) but not the regression test (p = 0.504).

#### 3.3.3. Prevalence of meropenem resistance.

A total of 353 isolates from 12 studies were analyzed, yielding a resistance proportion of 0.924 (95% CI, 0.817–0.970). Significant heterogeneity was observed (I² = 73.44%, p < 0.001). The fill-and-trim method adjusted the estimate to 0.880 (95% CI, 0.753–0.946). One study [67] was identified as a potential outlier. Upon exclusion, the proportion remained at 0.880 (95% CI, 0.753–0.946). There was no evidence of funnel plot asymmetry (p = 0.879 and p = 0.662, respectively).

#### 3.3.4. Prevalence of gentamicin resistance.

Among 356 isolates across 12 studies, the estimated resistance proportion was 0.777 (95% CI, 0.577–0.899) with significant heterogeneity (I² = 85.33%, p < 0.001). The fill-and-trim method adjusted the estimate to 0.557 (95% CI, 0.327–0.764). No outliers were detected. The regression test suggested funnel plot asymmetry (p = 0.002), while the rank correlation test did not (p = 0.191).

#### 3.3.5. Prevalence of sulfamethoxazole resistance.

From 360 isolates across 13 studies, the estimated resistance proportion was 0.461 (95% CI, 0.235–0.704), with significant heterogeneity (I² = 89.02%, p < 0.001). The fill-and-trim method adjusted the estimate to 0.537 (95% CI, 0.286–0.771). No outliers were detected. The rank correlation test (p = 0.765) or the regression test (p = 0.428) did not indicate Funnel plot asymmetry.

### 3.4. Summary of small-scale studies on *Elizabethkingia*

Smaller studies provided additional insights into the clinical impact of *Elizabethkingia* infections, resistance mechanisms, and treatment outcomes. Despite their limited sample sizes, these studies contribute valuable context to the broader resistance patterns identified. A summary of key findings is provided in the S2 Table in S1 File.

### 3.5. Subgroup and temporal analysis

Resistance rates for cefotaxime, ceftazidime, meropenem, and gentamicin was consistently high across studies. However, no significant differences were found across subgroups based on study year, region, species, or testing method (P3 > 0.05). The only notable variation was in sulfamethoxazole resistance, which ranged from 3.7% in Taiwan to 89.2% in India (p < 0.001) (S3 Table in S1 File).

Temporal Analysis showed no statistical differences in the resistance rates over time (S1 Fig).

## 4. Discussion

This systematic review and meta-analysis aimed to elucidate antibiotic resistance patterns in *Elizabethkingia*, a genus of increasing clinical importance. Following a thorough search of major databases (Scopus, PubMed, EMBASE, and Web of Science), we identified 1,016 studies, from which 34 met our inclusion criteria. These studies, published between 1998 and 2023, spanned seven countries across four continents, providing a global perspective on *Elizabethkingia* resistance.

The high prevalence of Ceftazidime resistance (88.5%, 95% CI: 21.1%–99.5%) in *Elizabethkingia* is alarming, especially given its role as a last-resort antibiotic for many gram-negative infections. Our analysis revealed a consistent resistance pattern, as indicated by the lack of significant heterogeneity among studies, suggesting a robust and entrenched resistance mechanism within the genus. However, as reflected by the broad 95% confidence interval, the strikingly high resistance rate shows a high degree of uncertainty in the pooled estimate. This suggests that the true resistance rate may be overestimated due to variability in study sample sizes, methodologies, and reporting practices. Despite this uncertainty, the lack of significant heterogeneity still points to an underlying resistance mechanism within the *Elizabethkingia* genus.

The potential outliers identified in the analysis highlight the need for cautious interpretation and consideration of individual study contexts. This high resistance rate underscores the urgent need for alternative treatment strategies and ongoing surveillance of antibiotic resistance. Nevertheless, further large-scale studies are needed to refine this estimate and confirm its clinical implications.

The high resistance rate to cefotaxime (96.1%) and meropenem (92.4%) further underscores the growing challenge in treating *Elizabethkingia* infections, particularly in the context of multi-drug-resistant (MDR) strains.

Interestingly, rifampin demonstrated a relatively lower resistance rate (12.5%), but the significant heterogeneity suggests regional variations or differing resistance mechanisms. Similarly, ciprofloxacin resistance was found in 27.9% of isolates, with notable variability across studies, suggesting that resistance mechanisms may vary by region or strain type. Gentamicin resistance (77.7%) also demonstrated high prevalence, though significant heterogeneity was observed. Differences may influence this variability in local antibiotic usage practices or strain genetic diversity. Sulfamethoxazole resistance was found in 46.1% of isolates, with heterogeneity suggesting that local factors, such as antibiotic use patterns and strain variability, contribute to resistance.

Our subgroup and analysis across time provide valuable insights into the complex and evolving landscape of antibiotic resistance in *Elizabethkingia* spp. The high resistance rates observed to critical antibiotics such as Cefotaxime, Ceftazidime, Meropenem, and Gentamicin are of significant concern, particularly in clinical settings where these antibiotics are commonly used to treat infections caused by Gram-negative bacteria. These findings highlight the urgent need for alternative treatment strategies and the development of new therapeutic options. However, no statistical differences were noted in the resistance rates over time. This lack of statistical significance should not be interpreted as evidence of true uniformity in resistance patterns. Several factors may contribute to this outcome. First, small sample sizes and underpowering of the analysis could mask real differences in resistance rates across time periods. The inclusion of studies with varying methodologies, including differences in study design, geographic scope, and sample populations, may also obscure trends.

Additionally, variations in how antibiotic resistance data are reported, including differences in the reporting thresholds and methods for susceptibility testing, could introduce inconsistencies that affect the ability to detect temporal changes. Another potential reason for the lack of statistical significance is the aggregated nature of the data in this meta-analysis, which combines diverse regions, species, and methods. This broad aggregation may dilute trends that exist within more specific subgroups, such as particular *Elizabethkingia* species or regional populations, where resistance patterns may vary more significantly. Furthermore, the relatively short duration of some studies may not provide enough time to observe significant shifts in resistance patterns, particularly if resistance development is a gradual process influenced by long-term factors such as selective pressure from antibiotics or evolving environmental conditions.

Finally, the lack of observed variation may also reflect the stability of resistance patterns in *Elizabethkingia*, suggesting that resistance mechanisms may be well-established and remain consistent over time. While this stability could be seen as positive in predictability for clinical treatment, it also highlights the persistent nature of antibiotic resistance in *Elizabethkingia* spp., which is difficult to overcome without new therapeutic strategies. Our subgroup analyses revealed key insights into regional and temporal resistance trends. Notably, the geographical variation in sulfamethoxazole resistance—high in India (89.2%) and low in Taiwan (3.7%)—emphasizes the importance of region-specific resistance data and suggests that local healthcare practices and strain diversity play a major role in resistance development. Additionally, meta-regression analysis did not reveal a clear temporal trend in resistance, suggesting that although resistance remains high, it has not worsened significantly over time. This stability may reflect the entrenched nature of resistance mechanisms across *Elizabethkingia* strains.

Whole-genome sequencing (WGS) has provided valuable insights into the antibiotic resistance mechanisms and global transmission potential of *Elizabethkingia* spp. Several studies have characterized the genomic diversity of *Elizabethkingia*, revealing a wide array of antibiotic resistance genes, mobile genetic elements, and horizontal gene transfer events that contribute to its multidrug resistance. Notably, Breurec et al. (2016) conducted a comparative genomic analysis of *Elizabethkingia* anophelis isolates from different geographical regions, demonstrating significant genomic plasticity and the presence of resistance determinants associated with β-lactams, fluoroquinolones, and aminoglycosides [68]. Similarly, Wang et al. (2019) identified mutations in topoisomerase genes (*gyrA* and *parC*), which correlate with fluoroquinolone resistance, further emphasizing the role of genetic adaptations in antibiotic resistance [69].

Teo et al. identified E. anopheles in the midgut of Anopheles mosquitoes, suggesting that these insects may act as vectors for environmental transmission, raising alarms about possible reservoirs in natural habitats [70]. Hem et al. further corroborated this by demonstrating that *Elizabethkingia* species are prevalent in aquatic environments, which could facilitate their spread in hospital settings [4].

The presence of plasmid-mediated resistance genes and integrative conjugative elements in *Elizabethkingia* species indicate a capacity for horizontal gene transfer, complicating infection control efforts [71]. Larkin et al. emphasized the importance of distinguishing between E. anophelis and other species due to their differing resistance profiles, which can influence treatment outcomes [72]. Continued genomic studies are essential for understanding the epidemiology and transmission dynamics of *Elizabethkingia* spp., particularly in healthcare settings.

In addition to large-scale studies, smaller-scale case reports have contributed valuable insights into the clinical implications of *Elizabethkingia* infections. For instance, Johny et al. reported a case of septicemia and meningitis in a six-month-old due to *E. meningosepticum*, highlighting discrepancies between disc diffusion and MIC results in antimicrobial susceptibility testing [73]. Bellais et al. (2000) discovered a carbapenem-hydrolyzing beta-lactamase gene in E. *meningosepticum*, further indicating a clinical threat [74]. Studies on nosocomial pneumonia in infants post-cardiac surgery [75] and catheter-related bacteremia in patients with chronic renal failure [76] underscore the clinical relevance of *E. meningosepticum*. Despite smaller sample sizes, these case reports offer crucial insights into the pathogen’s resistance profiles and highlight the urgent need for new therapeutic strategies. The consistent resistance patterns across different regions and settings point to entrenched resistance mechanisms that may be evolving, emphasizing the necessity of developing new antibiotics or alternative therapies [77].

While this meta-analysis offers significant insights into the global antibiotic resistance patterns of *Elizabethkingia*, several limitations must be acknowledged. First, the reliance on observational data introduces inherent biases, including potential publication bias and variability in study design, sample size, and laboratory methodologies. Despite our efforts to standardize resistance data using internationally recognized breakpoints (CLSI and EUCAST), differences in susceptibility testing protocols across studies could contribute to measurement inconsistencies. Furthermore, excluding non-English studies and case reports may limit the generalizability of our findings, particularly in regions where these types of studies are more common. Although subgroup and temporal analyses were conducted, the lack of statistical significance in some comparisons, such as by geographic region and testing method, may reflect insufficient sample sizes or underpowered subgroups. Finally, while the fill-and-trim method was employed to adjust for potential publication bias, the possibility of unpublished or underreported studies influencing the overall conclusions cannot be entirely ruled out. These limitations should be considered when interpreting the results, and further large-scale, standardized studies are needed to validate and refine these findings.

## 5. Conclusion

This systematic review and meta-analysis provide a comprehensive assessment of *Elizabethkingia* antibiotic resistance patterns, revealing high resistance rates to key antibiotics, including cefotaxime (96.1%), meropenem (92.4%), and ceftazidime (88.5%). These findings indicate widespread and entrenched resistance mechanisms across regions and study periods. While resistance to rifampin (12.5%) and ciprofloxacin (27.9%) was lower, significant heterogeneity suggests regional variations in resistance patterns. The substantial differences in sulfamethoxazole resistance, ranging from 3.7% in Taiwan to 89.2% in India, further emphasize the need for localized treatment strategies. Additionally, the lack of a clear increasing or decreasing trend in resistance over time, as identified in the meta-regression analysis, suggests that resistance levels remain persistently high. These results highlight the urgent need for new therapeutic options, standardized susceptibility testing, and continuous surveillance to mitigate the spread of *Elizabethkingia* resistance. By aligning the conclusion with the study’s findings, this research underscores the necessity of global efforts to address this emerging nosocomial pathogen.

## Supporting information

S1 File S1 Table: Comprehensive Data and Characteristics Extracted from Included Articles. S2 Table: JBI Checklist: Quality Assessment Results for Each Included Study. S3 Table: Meta-analysis statistics of worldwide antibiotic resistance in *Elizabethkingia*.
(DOCX)

S2 File Whole selection prosces. (XLSX)

S1 Fig Meta-regression analysis of antibiotic resistance trends over time for selected antibiotics. Plots A through D represent the proportion of resistance found in studies for Rifampin, Ciprofloxacin, Gentamicin, and Sulfamethoxazole, respectively, from 1998 to 2023. Each circle denotes individual research, with the size indicating its relative weight. Solid lines illustrate the 95% confidence interval prediction of the resistance proportion, whereas dashed lines show the confidence intervals for each study. The analyses revealed no statistically significant trends in resistance to these antibiotics over time, as indicated by the p-values and confidence intervals.
(JPEG)
