# Supplementary material for: Unraveling the global landscape of Elizabethkingia antibiotic resistance: A systematic review and meta-analysis
Source: PLoS One. 2025 May 30;20(5):e0323313. doi: 10.1371/journal.pone.0323313 (PMC12124755; doi:10.1371/journal.pone.0323313)
Supplement: S1 File — S2 Table: JBI Checklist: Quality Assessment Results for Each Included Study. S3 Table: Meta-analysis statistics of worldwide antibiotic resistance in Elizabethkingia. (DOCX) [file pone.0323313.s001.docx]

| **Section and Topic** | **Item #** | **Checklist item** | **Location where item is reported** |
| --- | --- | --- | --- |
| **TITLE** | | |  |
| Title | 1 | Identify the report as a systematic review. | Page 1 |
| **ABSTRACT** | | |  |
| Abstract | 2 | See the PRISMA 2020 for Abstracts checklist. | Page 2 |
| **INTRODUCTION** | | |  |
| Rationale | 3 | Describe the rationale for the review in the context of existing knowledge. | Page 3 |
| Objectives | 4 | Provide an explicit statement of the objective(s) or question(s) the review addresses. | Page 4 |
| **METHODS** | | |  |
| Eligibility criteria | 5 | Specify the inclusion and exclusion criteria for the review and how studies were grouped for the syntheses. | Page 4 and 5 |
| Information sources | 6 | Specify all databases, registers, websites, organisations, reference lists and other sources searched or consulted to identify studies. Specify the date when each source was last searched or consulted. | Page 6 |
| Search strategy | 7 | Present the full search strategies for all databases, registers and websites, including any filters and limits used. | Page 6, Lines 91-96 |
| Selection process | 8 | Specify the methods used to decide whether a study met the inclusion criteria of the review, including how many reviewers screened each record and each report retrieved, whether they worked independently, and if applicable, details of automation tools used in the process. | Page 6, Lines 97-99 |
| Data collection process | 9 | Specify the methods used to collect data from reports, including how many reviewers collected data from each report, whether they worked independently, any processes for obtaining or confirming data from study investigators, and if applicable, details of automation tools used in the process. | Page 6, Lines 100-104 |
| Data items | 10a | List and define all outcomes for which data were sought. Specify whether all results that were compatible with each outcome domain in each study were sought (e.g. for all measures, time points, analyses), and if not, the methods used to decide which results to collect. | Page 7, Lines 105-107 |
|  | 10b | List and define all other variables for which data were sought (e.g. participant and intervention characteristics, funding sources). Describe any assumptions made about any missing or unclear information. | Page 7, Lines 105-107 |
| Study risk of bias assessment | 11 | Specify the methods used to assess risk of bias in the included studies, including details of the tool(s) used, how many reviewers assessed each study and whether they worked independently, and if applicable, details of automation tools used in the process. | Page 7, Lines 108-114 |
| Effect measures | 12 | Specify for each outcome the effect measure(s) (e.g. risk ratio, mean difference) used in the synthesis or presentation of results. | Page 7, Lines 115-118 |
| Synthesis methods | 13a | Describe the processes used to decide which studies were eligible for each synthesis (e.g. tabulating the study intervention characteristics and comparing against the planned groups for each synthesis (item #5)). | Page 7and 8, Lines 119-124 |
|  | 13b | Describe any methods required to prepare the data for presentation or synthesis, such as handling of missing summary statistics, or data conversions. | Page 7and 8, Lines 119-124 |
|  | 13c | Describe any methods used to tabulate or visually display results of individual studies and syntheses. | Page 7and 8, Lines 119-124 |
|  | 13d | Describe any methods used to synthesize results and provide a rationale for the choice(s). If meta-analysis was performed, describe the model(s), method(s) to identify the presence and extent of statistical heterogeneity, and software package(s) used. | Page 7and 8, Lines 119-124 |
|  | 13e | Describe any methods used to explore possible causes of heterogeneity among study results (e.g. subgroup analysis, meta-regression). | Page 7and 8, Lines 119-124 |
|  | 13f | Describe any sensitivity analyses conducted to assess robustness of the synthesized results. | Page 7and 8, Lines 119-124 |
| Reporting bias assessment | 14 | Describe any methods used to assess risk of bias due to missing results in a synthesis (arising from reporting biases). | Lines 130-133 |
| Certainty assessment | 15 | Describe any methods used to assess certainty (or confidence) in the body of evidence for an outcome. | Lines 130-133 |
| **RESULTS** | | |  |
| Study selection | 16a | Describe the results of the search and selection process, from the number of records identified in the search to the number of studies included in the review, ideally using a flow diagram. | Lines 135-141 |
|  | 16b | Cite studies that might appear to meet the inclusion criteria, but which were excluded, and explain why they were excluded. | Figure 1 |
| Study characteristics | 17 | Cite each included study and present its characteristics. | Supplementary Table 1 |
| Risk of bias in studies | 18 | Present assessments of risk of bias for each included study. |  |
| Results of individual studies | 19 | For all outcomes, present, for each study: (a) summary statistics for each group (where appropriate) and (b) an effect estimates and its precision (e.g. confidence/credible interval), ideally using structured tables or plots. | Supplementary Table 1 |
| Results of syntheses | 20a | For each synthesis, briefly summarise the characteristics and risk of bias among contributing studies. | Lines 148-160 |
|  | 20b | Present results of all statistical syntheses conducted. If meta-analysis was done, present for each the summary estimate and its precision (e.g. confidence/credible interval) and measures of statistical heterogeneity. If comparing groups, describe the direction of the effect. | Lines 161-220 |
|  | 20c | Present results of all investigations of possible causes of heterogeneity among study results. | Lines 161-220 |
|  | 20d | Present results of all sensitivity analyses conducted to assess the robustness of the synthesized results. | Lines 161-220 |
| Reporting biases | 21 | Present assessments of risk of bias due to missing results (arising from reporting biases) for each synthesis assessed. | Lines 161-220 |
| Certainty of evidence | 22 | Present assessments of certainty (or confidence) in the body of evidence for each outcome assessed. | Lines 161-220 |
| **DISCUSSION** | | |  |
| Discussion | 23a | Provide a general interpretation of the results in the context of other evidence. | Lines 221-312 |
|  | 23b | Discuss any limitations of the evidence included in the review. | Lines 221-312 |
|  | 23c | Discuss any limitations of the review processes used. | Lines 221-312 |
|  | 23d | Discuss implications of the results for practice, policy, and future research. | Lines 221-312 |
| **OTHER INFORMATION** | | |  |
| Registration and protocol | 24a | Provide registration information for the review, including register name and registration number, or state that the review was not registered. | Line 37-39 |
|  | 24b | Indicate where the review protocol can be accessed, or state that a protocol was not prepared. | Line 37-39 |
|  | 24c | Describe and explain any amendments to information provided at registration or in the protocol. | Line 37-39 |
| Support | 25 | Describe sources of financial or non-financial support for the review, and the role of the funders or sponsors in the review. | Line 343 |
| Competing interests | 26 | Declare any competing interests of review authors. | Line 345-346 |
| Availability of data, code and other materials | 27 | Report which of the following are publicly available and where they can be found: template data collection forms; data extracted from included studies; data used for all analyses; analytic code; any other materials used in the review. | Supplementary File |

## The search syntax was used for the literature review in the PubMed database.

(“*Elizabethkingia*” OR “*Elizabethkingia* anopheles” OR "E. anopheles” OR “Flavobacterium anhuiense” OR “F. anhuiense” OR “*Elizabethkingia* *meningoseptica*” OR “E. meningoseptica” OR “Chryseobacterium anopheles”) AND (“antibiotic resistan*” OR “antimicrobial resistan*” OR “drug resistan*” OR “antibiotic susceptibility” OR “multi-drug resistan*” OR “MDR” OR “pan-drug resistan*” OR “PDR” OR “extensively drug-resistant” OR “XDR” OR “antibiotic therapy” OR “antibiotic treatment” OR “antimicrobial therapy” OR “antimicrobial stewardship” OR “antimicrobial susceptibility” OR “beta-lactamase production” OR “carbapenem resistance” OR “antibiotic sensitivity” OR “resistance mechanisms” OR “efflux pump inhibitors” OR “penicillin-binding proteins” OR “aminoglycoside resistan*” OR “antibiotic degradation” OR “horizontal gene transfer” OR “plasmid-mediated resistan*” OR “quinolone resistan*” OR “antibiotic efflux” OR “antibiotic stewardship” OR “resistant strains” OR “antibiotic selective pressure” OR “surveillance studies” OR “therapeutic guidelines”)

## Supplementary Table 1: Comprehensive Data and Characteristics Extracted from Included Articles

| Author | AST classification | Species | Sample source | Country | Sample Size | Ceftazidime | Ampicillin | Amoxicillin | Vancomycin | Cefpodoxime | Chloramphenicol | Tetracycline | Rifampin | Ciprofloxacin | Ofloxacin | Enrofloxacin | Cefotaxime | Meropenem | Gentamicin | Sulfamethoxazole |
| --- | --- | --- | --- | --- | --- | --- | --- | --- | --- | --- | --- | --- | --- | --- | --- | --- | --- | --- | --- | --- |
| M. K. Johny (1983) (1) | M | *E. Meningoseptica* | C | Kuwait | 2 |  | 2 |  | 2 |  | 2 |  | 0 |  |  | 2 |  |  | 0 |  |
| M. C. M. Di Pentima (1998) (2) | M | *E. Meningoseptica* | C | USA | 4 |  |  |  | 4 |  |  |  |  | 0 |  |  |  | 4 |  | 0 |
| S. A. Bellais (2000) (3) | M | *E. Meningoseptica* | C | France | 1 | 1 |  |  |  |  |  |  |  |  |  |  | 1 | 1 |  |  |
| F. Emele (2000) (4) | I | *E. Meningoseptica* | C | Nigeria | 1 |  | 1 |  |  |  | 1 |  |  | 0 |  |  |  |  |  |  |
| S. Ö. Güngör (2003) (5) | I | *E. Meningoseptica* | C | Turkey | 4 | 4 |  |  | 0 | 0 |  |  |  | 0 |  |  | 4 |  |  |  |
| J. T. S. Kirby (2004) (6) | M | *E. Meningoseptica* | C | MC | 24 |  |  |  | 1 |  |  |  | 0 | 4 |  |  |  | 22 | 23 | 5 |
| L. H. S. Tan (2004) (7) |  | *E. Meningoseptica* | C | China | 1 | 1 |  |  | 1 | 1 |  |  |  |  |  |  | 1 | 1 |  | 1 |
| N. A. Ozkalay (2006) (8) | M | *E. Meningoseptica* | C | Turkey | 1 |  |  |  | 0 |  |  |  |  | 0 |  |  |  | 1 | 1 | 0 |
| Z. Y. Z. Xie (2009) (9) | I | *E. Meningoseptica* | A | China | 1 | 1 |  |  | 0 |  | 1 | 1 |  | 1 |  |  |  |  | 1 |  |
| A. I. S. Adeyemi (2010) (10) | I | *E. Meningoseptica* | C | Nigeria | 1 | 0 | 1 |  |  |  | 1 | 1 |  |  | 0 |  | 0 |  | 0 | 1 |
| P. M. Kaempfer (2011) (11) | M | *E. Anophelis* | A | Germany | 1 |  |  |  |  |  | 1 | 1 |  |  |  |  |  |  |  |  |
| X. W. Jiang (2012) (12) | M | *E. Meningoseptica* | A | China | 26 |  |  |  | 0 |  | 8 | 6 |  | 6 |  |  |  | 13 | 12 | 15 |
| V. M. Tak (2013) (13) | M | *E. Meningoseptica* | C | China | 7 |  |  |  |  | 7 |  |  |  | 3 |  |  |  |  | 7 | 6 |
| B. F. Bayrak (2014) (14) | I | *E. Meningoseptica* | C | Turkey | 1 |  |  |  | 0 |  |  |  | 1 | 0 |  |  |  | 1 | 1 | 0 |
| V. V. R. Shailaja (2014) (15) | M | *E. Meningoseptica* | C | India | 9 |  |  |  | 0 |  |  |  |  | 8 |  |  | 9 | 9 | 9 | 9 |
| B. W. A. Arega (2017) (16) | I | *E. Meningoseptica* | C | Ethiopia | 1 |  |  |  |  |  |  |  |  |  |  |  |  |  |  |  |
| H. S. H. EbrahimSaraie (2018) (17) | M | *E. Meningoseptica* | C | Iran | 1 |  |  |  | 0 |  |  |  |  | 0 |  |  |  | 1 |  | 0 |
| W. L. R. Johnson (2018) (18) | M | *E. Anophelis* | A | USA | 2 | 2 | 2 |  | 2 |  | 2 |  | 0 | 0 |  | 2 |  |  | 0 | 0 |
| T. Y. C. Chang (2019) (19) | M | *E. Meningoseptica* | C | Taiwan | 11 |  |  |  | 0 |  |  |  | 0 |  |  |  |  |  |  |  |
| T. Y. C. Chang (2019) (19) | M | *E. Miricola* | C | Taiwan | 14 |  |  |  | 3 |  |  |  | 3 |  |  |  |  |  |  |  |
| T. Y. C. Chang (2019) (19) | M | *E. Anophelis* | C | Taiwan | 142 |  |  |  | 6 |  |  |  | 8 |  |  |  |  |  |  |  |
| P. S. Joshi (2019) (20) | M | *E. Meningoseptica* | C | India | 1 |  |  |  | 1 |  |  |  | 0 | 0 |  |  |  |  |  |  |
| B. L. idenov (2019) (21) | M | *E. Anophelis* | C | Australia | 8 |  |  |  | 3 |  |  |  | 0 | 0 |  |  |  |  |  |  |
| B. L. idenov (2019) (21) | M | *E. Miricola* | C | Australia | 3 |  |  |  | 0 |  |  |  | 0 | 0 |  |  |  |  |  |  |
| B. L. idenov (2019) (21) | M | *E. Meningoseptica* | C | Australia | 3 |  |  |  | 3 |  |  |  | 0 | 0 |  |  |  |  |  |  |
| B. L. idenov (2019) (21) | M | *E. Bruunia* | C | Australia | 4 |  |  |  | 2 |  |  |  | 0 | 0 |  |  |  |  |  |  |
| B. L. idenov (2019) (21) | M | *E. Ursingii* | C | Australia | 2 |  |  |  | 0 |  |  |  | 0 | 0 |  |  |  |  |  |  |
| B. L. idenov (2019) (21) | M | *E. Occulta* | C | Australia | 1 |  |  |  | 1 |  |  |  | 0 | 0 |  |  |  |  |  |  |
| A. I. K. Barwi (2020) (22) |  | *E. Meningoseptica* | C | Saudi Arabia | 1 | 1 |  |  |  |  |  |  |  |  |  |  | 1 | 1 |  | 1 |
| D. G. Burrd (2020) (23) | M | *E. Anophelis* | C | Australia | 16 | 16 | 16 | 16 |  |  | 13 |  |  | 7 |  |  | 15 | 15 | 8 | 4 |
| D. G. Burrd (2020) (23) | M | *E. Meningoseptica* | C | Australia | 2 | 2 | 2 | 2 |  |  |  |  |  |  |  |  | 2 | 2 |  |  |
| D. G. Burrd (2020) (23) | M | *E. Miricola* | C | Australia | 3 | 3 |  |  |  |  |  |  |  |  |  |  | 3 | 2 | 2 | 2 |
| R. Z. Hu (2020) (24) | M | *E. Miricola* | C | China | 1 | 1 | 1 | 1 |  |  | 1 | 1 | 1 | 1 |  |  | 1 | 1 | 1 | 1 |
| G. C. A. L. Johnston (2020) (25) | I | *E. Meningoseptica* | A | Australia | 1 | 1 | 1 |  |  |  |  | 1 |  |  |  | 0 |  |  | 1 | 1 |
| H. K. Seong (2020) (26) | M | *E. Anophelis* | C | Korea | 44 |  |  |  | 3 |  |  |  | 25 |  |  |  |  |  |  |  |
| H. K. Seong (2020) (26) | M | *E. Miricola* | C | Korea | 17 |  |  |  | 17 |  |  |  | 1 |  |  |  |  |  |  |  |
| H. K. Seong (2020) (26) | M | *E. Meningoseptica* | C | Korea | 149 |  |  |  | 0 |  |  |  | 0 |  |  |  |  |  |  |  |
| L. Z. Wang (2020) (27) | M | *Elizabethkingia Spp.* | C | China | 52 | 52 |  |  | 40 |  |  |  | 6 | 20 |  |  |  | 48 | 50 | 33 |
| A. R. C. da Costa (2021) (28) | I | *E. Meningoseptica* | A | Brazil | 1 |  |  |  |  |  |  | 1 |  |  |  | 1 |  |  | 0 |  |
| S. C. T. Kuo (2021) (29) | M | *E. Anophelis* | C | Taiwan | 90 |  |  |  | 90 |  |  |  | 17 | 85 |  |  | 90 | 90 | 89 | 3 |
| S. C. T. Kuo (2021) (29) | M | *E. Meningoseptica* | C | Taiwan | 7 |  |  |  | 7 |  |  |  | 0 | 40 |  |  | 7 | 7 | 7 | 0 |
| S. C. T. Kuo (2021) (29) | M | *E. Miricola* | C | Taiwan | 11 |  |  |  | 11 |  |  |  | 8 | 5 |  |  | 11 | 11 | 2 | 0 |
| L. P. Xu (2021) (30) | M | *E. Anophelis* | A | China | 31 | 31 | 31 |  |  |  |  | 31 |  | 2 |  |  |  | 31 | 21 | 28 |
| A. W. A. Hashmi (2023) (31) |  | *E. Meningoseptica* | C | Pakistan | 1 |  | 1 |  | 0 |  | 1 | 1 |  | 0 |  |  |  | 1 |  | 1 |
| S. W. Li (2023) (32) | M | *E. Miricola* | A | China | 1 |  |  |  |  |  |  |  |  |  |  | 1 |  |  |  | 1 |
| S. B. Sarathi (2023) (33) | I | *Elizabethkingia Spp.* | C | India | 80 | 2 | 2 |  | 1 |  | 7 |  |  | 5 |  |  | 78 | 77 | 27 | 71 |
| D. C. Wei (2023) (34) | I | *E. Miricola* | A | China | 1 |  |  | 1 |  |  |  |  | 0 |  |  | 0 |  |  | 1 | 1 |

Abbreviations: Sample (C: Clinical, A: Animal); AST classification (M: Minimum Inhibitor Concentration Methods, I; Inhibition Zone Methods)

## Supplementary Table 2: JBI Checklist: Quality Assessment Results for Each Included Study

| Author (Year) | Were the criteria for inclusion in the sample clearly defined? | Were the study subjects and the setting described in detail? | Were objective, standard criteria used for measurement of the condition? | Were confounding factors identified? | Were strategies to deal with confounding factors stated? | Were the outcomes measured in a valid and reliable way? | Was appropriate statistical analysis used? |
| --- | --- | --- | --- | --- | --- | --- | --- |
| M. K. Johny (1983) (1) | L | L | L | L | L | L | L |
| M. C. M. Di Pentima (1998) (2) | L | L | L | L | L | L | L |
| S. A. Bellais (2000) (3) | L | L | L | L | L | L | L |
| F. Emele (2000) (4) | L | L | L | L | L | L | L |
| S. Ö. Güngör (2003) (5) | L | L | L | L | L | L | L |
| J. T. S. Kirby (2004) (6) | L | L | L | L | L | L | L |
| L. H. S. Tan (2004) (7) | L | L | L | L | L | L | L |
| N. A. Ozkalay (2006) (8) | L | L | L | L | L | L | L |
| Z. Y. Z. Xie (2009) (9) | L | L | L | L | L | L | L |
| A. I. S. Adeyemi (2010) (10) | L | L | L | L | L | L | L |
| P. M. Kaempfer (2011) (11) | L | L | H | L | L | L | L |
| X. W. Jiang (2012) (12) | L | L | L | L | L | L | L |
| V. M. Tak (2013) (13) | L | L | L | L | L | L | L |
| B. F. Bayrak (2014) (14) | L | L | L | L | L | L | L |
| V. V. R. Shailaja (2014) (15) | L | L | L | L | L | L | L |
| B. W. A. Arega (2017) (16) | L | L | L | L | L | L | L |
| H. S. H. EbrahimSaraie (2018) (17) | L | L | L | L | L | L | L |
| W. L. R. Johnson (2018) (18) | L | L | L | L | L | L | L |
| T. Y. C. Chang (2019) (19) | L | L | L | L | L | L | L |
| T. Y. C. Chang (2019) (19) | L | L | L | L | L | L | L |
| T. Y. C. Chang (2019) (19) | L | L | L | L | L | L | L |
| P. S. Joshi (2019) (20) | L | L | L | L | L | L | L |
| B. L. idenov (2019) (21) | L | L | L | L | L | L | L |
| B. L. idenov (2019) (21) | L | L | L | L | L | L | L |
| B. L. idenov (2019) (21) | L | L | L | L | L | L | L |
| B. L. idenov (2019) (21) | L | L | L | L | L | L | L |
| B. L. idenov (2019) (21) | L | L | L | L | L | L | L |
| B. L. idenov (2019) (21) | L | L | L | L | L | L | L |
| A. I. K. Barwi (2020) (22) | L | L | H | L | L | H | L |
| D. G. Burrd (2020) (23) | L | L | L | L | L | L | L |
| D. G. Burrd (2020) (23) | L | L | L | L | L | L | L |
| D. G. Burrd (2020) (23) | L | L | L | L | L | L | L |
| R. Z. Hu (2020) (24) | L | L | L | L | L | L | L |
| G. C. A. L. Johnston (2020) (25) | L | L | L | L | L | L | L |
| H. K. Seong (2020) (26) | L | L | L | L | L | L | L |
| H. K. Seong (2020) (26) | L | L | L | L | L | L | L |
| H. K. Seong (2020) (26) | L | L | L | L | L | L | L |
| L. Z. Wang (2020) (27) | L | L | L | L | L | L | L |
| A. R. C. da Costa (2021) (28) | H | H | L | L | L | L | L |
| S. C. T. Kuo (2021) (29) | L | L | L | L | L | L | L |
| S. C. T. Kuo (2021) (29) | L | L | L | L | L | L | L |
| S. C. T. Kuo (2021) (29) | L | L | L | L | L | L | L |
| L. P. Xu (2021) (30) | L | L | L | L | L | L | L |
| A. W. A. Hashmi (2023) (31) | L | L | H | L | L | H | L |
| S. W. Li (2023) (32) | L | L | L | L | L | L | L |
| S. B. Sarathi (2023) (33) | L | L | L | L | L | L | L |
| D. C. Wei (2023) (34) | L | L | L | L | L | L | L |

Abbreviations: JBI checklist of Quality assessment questions (L: Low Risk, H: High Risk)

## Supplementary Table 3: Meta-analysis statistics of worldwide antibiotic resistance in *Elizabethkingia*

| Antibiotic | Category | Subgroup | K (n, N) | Proportion 95%CI | I² | P1 | P2 | | P3 |
| --- | --- | --- | --- | --- | --- | --- | --- | --- | --- |
| Ceftazidim | Overall | NA | 5 (108, 186) | 0.885 (0.211, 0.995) | 0.00% | p<0.001 | p=0.623 | | NA |
| Rifampin | Overall | NA | 15 (68, 579) | 0.125 (0.059, 0.247) | 82.01% | p<0.001 | p<0.001 | | NA |
|  | Year group | 1998-2019 | 1 (0, 24) | 0.020 (0.001, 0.251) | 0.00% | p=0.006 | p>0.999 | | p=0.298 |
|  |  | 2020-2023 | 14 (68, 555) | 0.137 (0.064, 0.269) | 82.59% | p<0.001 | p<0.001 | |  |
|  | Countries | Taiwan | 6 (36, 275) | 0.173 (0.065, 0.389) | 81.99% | p=0.006 | p<0.001 | | p=0.946 |
|  |  | Australia | 4 (0, 18) | 0.096 (0.024, 0.314) | 0.00% | p=0.003 | p=0.970 | |  |
|  |  | South Korea | 3 (26, 210) | 0.074 (0.003, 0.715) | 91.57% | p=0.151 | p<0.001 | |  |
|  |  | China | 1 (6, 52) | 0.115 (0.053, 0.234) | 0.00% | p<0.001 | p>0.999 | |  |
|  | Continents | NA | 1 (0, 24) | 0.020 (0.001, 0.251) | 0.00% | p=0.006 | p>0.999 | | p=0.529 |
|  |  | Asia | 10 (68, 537) | 0.146 (0.061, 0.311) | 87.66% | p<0.001 | p<0.001 | |  |
|  |  | Oceania | 4 (0, 18) | 0.096 (0.024, 0.314) | 0.00% | p=0.003 | p=0.970 | |  |
|  | Species | *E. Meningoseptica* | 5 (0, 194) | 0.029 (0.008, 0.095) | 0.00% | p<0.001 | p=0.420 | | p=0.347 |
|  |  | *E. Miricola* | 4 (12, 45) | 0.251 (0.056, 0.652) | 74.41% | p=0.213 | p=0.008 | |  |
|  |  | *E. Anophelis* | 4 (50, 284) | 0.176 (0.045, 0.491) | 93.39% | p=0.045 | p<0.001 | |  |
|  |  | *E. Bruuniana* | 1 (0, 4) | 0.100 (0.006, 0.674) | 0.00% | p=0.140 | p>0.999 | |  |
|  |  | *Elizabethkingia Spp.* | 1 (6, 52) | 0.115 (0.053, 0.234) | 0.00% | p<0.001 | p>0.999 | |  |
| Ciprofloxacin | Overall | NA | 15 (145, 372) | 0.279 (0.138, 0.484) | 66.15% | p<0.001 | p<0.001 | | NA |
|  | Year group | 1998-2019 | 6 (21, 74) | 0.300 (0.133, 0.544) | 58.40% | p=0.104 | p=0.035 | | p=0.849 |
|  |  | 2020-2023 | 10 (124, 298) | 0.259 (0.090, 0.550) | 90.04% | p=0.100 | p<0.001 | |  |
|  | Countries | US | 1 (0, 4) | 0.100 (0.006, 0.674) | 0.00% | p=0.140 | p>0.999 | | p=0.282 |
|  |  | Turkey | 1 (0, 4) | 0.100 (0.006, 0.674) | 0.00% | p=0.140 | p>0.999 | |  |
|  |  | China | 4 (31, 116) | 0.256 (0.124, 0.455) | 67.05% | p=0.018 | p=0.028 | |  |
|  |  | India | 2 (13, 89) | 0.399 (0.006, 0.986) | 94.16% | p=0.864 | p<0.001 | |  |
|  |  | Australia | 5 (7, 34) | 0.225 (0.087, 0.470) | 22.07% | p=0.030 | p=0.274 | |  |
|  |  | Taiwan | 2 (90, 101) | 0.794 (0.167, 0.987) | 93.64% | p=0.370 | p<0.001 | |  |
|  | Continents | Americas | 1 (0, 4) | 0.100 (0.006, 0.674) | 0.00% | p=0.140 | p>0.999 | | p=0.680 |
|  |  | Asia | 9 (134, 310) | 0.376 (0.146, 0.679) | 91.49% | p=0.428 | p<0.001 | |  |
|  |  | NA | 1 (4, 24) | 0.167 (0.064, 0.369) | 0.00% | p=0.003 | p>0.999 | |  |
|  |  | Oceania | 5 (7, 34) | 0.225 (0.087, 0.470) | 22.07% | p=0.030 | p=0.274 | |  |
|  | AST CLASSIFICATION | MIC | 14 (140, 288) | 0.329 (0.167, 0.545) | 83.82% | p=0.118 | p<0.001 | | p=0.157 |
|  |  | Inhibition Zone | 2 (5, 84) | 0.065 (0.029, 0.142) | 0.00% | p<0.001 | p=0.743 | |  |
|  | Species | *E. Meningoseptica* | 7 (21, 77) | 0.281 (0.133, 0.501) | 51.57% | p=0.051 | p=0.054 | | p=0.939 |
|  |  | *E. Anophelis* | 4 (94, 145) | 0.357 (0.037, 0.890) | 94.23% | p=0.667 | p<0.001 | |  |
|  |  | *E. Miricola* | 2 (5, 14) | 0.373 (0.132, 0.698) | 14.72% | p=0.454 | p=0.279 | |  |
|  |  | *E. Bruuniana* | 1 (0, 4) | 0.100 (0.006, 0.674) | 0.00% | p=0.140 | p>0.999 | |  |
|  |  | *Elizabethkingia Spp.* | 2 (25, 132) | 0.174 (0.023, 0.653) | 94.12% | p=0.163 | p<0.001 | |  |
|  | Sample | Clinical | 14 (137, 315) | 0.311 (0.143, 0.549) | 86.19% | p=0.116 | p<0.001 | | p=0.391 |
|  |  | Animal | 2 (8, 57) | 0.138 (0.037, 0.400) | 65.25% | p=0.012 | p=0.090 | |  |
| Cefotaxime | Overall | NA | 8 (217, 220) | 0.961 (0.916, 0.983) | 0.00% | p<0.001 | p=0.789 | | NA |
| Meropenem | Overall | NA | 11 (329, 353) | 0.924 (0.817, 0.970) | 0.00% | p<0.001 | p=0.612 | | NA |
| Gentamicin | Overall | NA | 12 (257, 356) | 0.777 (0.577, 0.899) | 85.33% | p=0.009 | p<0.001 | | NA |
|  | Year group | 1998-2019 | 4 (51, 66) | 0.876 (0.451, 0.984) | 79.33% | p=0.075 | p=0.002 | | p=0.460 |
|  |  | 2020-2023 | 8 (206, 290) | 0.736 (0.461, 0.901) | 88.12% | p=0.089 | p<0.001 | |  |
|  | Countries | China | 4 (90, 116) | 0.798 (0.485, 0.943) | 84.04% | p=0.061 | p<0.001 | | p=0.843 |
|  |  | India | 2 (36, 89) | 0.701 (0.066, 0.987) | 83.50% | p=0.634 | p=0.014 | |  |
|  |  | Australia | 2 (10, 19) | 0.525 (0.308, 0.732) | 0.00% | p=0.831 | p=0.600 | |  |
|  |  | Taiwan | 3 (98, 108) | 0.863 (0.096, 0.997) | 91.55% | p=0.377 | p<0.001 | |  |
|  | Continents | NA | 1 (23, 24) | 0.958 (0.756, 0.994) | 0.00% | p=0.002 | p>0.999 | | p=0.408 |
|  |  | Asia | 9 (224, 313) | 0.787 (0.542, 0.920) | 87.90% | p=0.024 | p<0.001 | |  |
|  |  | Oceania | 2 (10, 19) | 0.525 (0.308, 0.732) | 0.00% | p=0.831 | p=0.600 | |  |
|  | AST Classification | MIC | 11 (230, 276) | 0.818 (0.618, 0.926) | 81.02% | p=0.004 | p<0.001 | | p=0.163 |
|  |  | Inhibition Zone | 1 (27, 80) | 0.338 (0.243, 0.447) | 0.00% | p=0.004 | p>0.999 | |  |
|  | Species | *E. Meningoseptica* | 5 (58, 73) | 0.887 (0.548, 0.981) | 75.85% | p=0.031 | p=0.002 | | p=0.553 |
|  |  | *E. Anophelis* | 3 (118, 137) | 0.817 (0.418, 0.965) | 87.53% | p=0.109 | p<0.001 | |  |
|  |  | *E. Miricola* | 2 (4, 14) | 0.353 (0.062, 0.819) | 56.27% | p=0.574 | p=0.130 | |  |
|  |  | *Elizabethkingia Spp.* | 2 (77, 132) | 0.771 (0.069, 0.993) | 96.20% | p=0.533 | p<0.001 | |  |
|  | Sample | Clinical | 10 (224, 299) | 0.833 (0.570, 0.949) | 87.50% | p=0.017 | p<0.001 | | p=0.340 |
|  |  | Animal | 2 (33, 57) | 0.574 (0.359, 0.764) | 62.34% | p=0.506 | p=0.103 | |  |
| Sulfamethoxazole | Overall | NA | 13 (176, 360) | 0.461 (0.235, 0.704) | 89.02% | p=0.765 | p<0.001 | | NA |
|  | Year group | 1998-2019 | 5 (35, 70) | 0.542 (0.223, 0.830) | 75.83% | p=0.816 | p=0.002 | | p=0.594 |
|  |  | 2020-2023 | 8 (141, 290) | 0.399 (0.134, 0.740) | 92.21% | p=0.580 | p<0.001 | |  |
|  | Countries | US | 1 (0, 4) | 0.100 (0.006, 0.674) | 0.00% | p=0.140 | p>0.999 | | p<0.001 |
|  |  | China | 4 (82, 116) | 0.731 (0.542, 0.861) | 65.08% | p=0.019 | p=0.035 | |  |
|  |  | India | 2 (80, 89) | 0.892 (0.809, 0.942) | 0.00% | p<0.001 | p=0.556 | |  |
|  |  | Australia | 2 (6, 19) | 0.371 (0.103, 0.752) | 42.89% | p=0.527 | p=0.186 | |  |
|  |  | Taiwan | 3 (3, 108) | 0.037 (0.014, 0.095) | 0.00% | p<0.001 | p=0.912 | |  |
|  | Continents | Americas | 1 (0, 4) | 0.100 (0.006, 0.674) | 0.00% | p=0.140 | p>0.999 | | p=0.661 |
|  |  | NA | 1 (5, 24) | 0.208 (0.089, 0.413) | 0.00% | p=0.008 | p>0.999 | |  |
|  |  | Asia | 9 (165, 313) | 0.548 (0.256, 0.811) | 90.72% | p=0.764 | p<0.001 | |  |
|  |  | Oceania | 2 (6, 19) | 0.371 (0.103, 0.752) | 42.89% | p=0.527 | p=0.186 | |  |
|  | AST CLASSIFICATION | MIC | 12 (105, 280) | 0.405 (0.196, 0.655) | 85.97% | p=0.461 | p<0.001 | | p=0.145 |
|  |  | Inhibition Zone | 1 (71, 80) | 0.887 (0.798, 0.940) | 0.00% | p<0.001 | p>0.999 | |  |
|  | Species | *E. Meningoseptica* | 6 (35, 77) | 0.456 (0.177, 0.766) | 74.48% | p=0.800 | p=0.001 | | p=0.550 |
|  |  | *E. Anophelis* | 3 (35, 137) | 0.321 (0.020, 0.917) | 95.49% | p=0.641 | p<0.001 | |  |
|  |  | *E. Miricola* | 2 (2, 14) | 0.241 (0.007, 0.931) | 75.53% | p=0.550 | p=0.043 | |  |
|  |  | *Elizabethkingia Spp.* | 2 (104, 132) | 0.785 (0.453, 0.941) | 90.91% | p=0.087 | p<0.001 | |  |
|  | Sample | Clinical | 11 (133, 303) | 0.384 (0.153, 0.681) | 89.82% | p=0.451 | p<0.001 | | p=0.236 |
|  |  | Animal | 2 (43, 57) | 0.772 (0.340, 0.957) | 85.77% | p=0.205 | p=0.008 | |  |
| Caption; K: Number of reports, n: Number of resistant isolates, N: Number of total isolates, LCI: 95% Lower Limit Confidence Interval, HCI: 95% Higher Limit Confidence Interval, P1: P-value of difference from zero resistance rate, P2: P-value of heterogeneity between reports, P3: P-value of difference between groups. | | | | | | | |  |  |

1. Johny M, Khuffash FA, Elhag KM. Antimicrobial treatment of Flavobacterium meningosepticum infection. Ann Trop Paediatr. 1983;3(3):125-8.

2. Di Pentima MC, Mason EO, Jr., Kaplan SL. In vitro antibiotic synergy against Flavobacterium meningosepticum: implications for therapeutic options. Clin Infect Dis. 1998;26(5):1169-76.

3. Bellais S, Aubert D, Naas T, Nordmann P. Molecular and biochemical heterogeneity of class B carbapenem-hydrolyzing beta-lactamases in Chryseobacterium meningosepticum. Antimicrob Agents Chemother. 2000;44(7):1878-86.

4. Emele FE. Etiologic spectrum and pattern of antimicrobial drug susceptibility in bacterial meningitis in Sokoto, Nigeria. Acta Paediatr. 2000;89(8):942-6.

5. Gungor S, Ozen M, Akinci A, Durmaz R. A Chryseobacterium meningosepticum outbreak in a neonatal ward. Infect Control Hosp Epidemiol. 2003;24(8):613-7.

6. Kirby JT, Sader HS, Walsh TR, Jones RN. Antimicrobial susceptibility and epidemiology of a worldwide collection of Chryseobacterium spp: report from the SENTRY Antimicrobial Surveillance Program (1997-2001). J Clin Microbiol. 2004;42(1):445-8.

7. Tan L, Sun X, Zhu X, Zhang Z, Li J, Shu Q. Epidemiology of nosocomial pneumonia in infants after cardiac surgery. Chest. 2004;125(2):410-7.

8. Ozkalay N, Anil M, Agus N, Helvaci M, Sirti S. Community-acquired meningitis and sepsis caused by Chryseobacterium meningosepticum in a patient diagnosed with thalassemia major. J Clin Microbiol. 2006;44(8):3037-9.

9. Xie ZY, Zhou YC, Wang SF, Mei B, Xu XD, Wen WY, et al. First isolation and identification of Elizabethkingia meningoseptica from cultured tiger frog, Rana tigerina rugulosa. Vet Microbiol. 2009;138(1-2):140-4.

10. Adeyemi AI, Sulaiman AA, Solomon BB, Chinedu OA, Victor IA. Bacterial bloodstream infections in HIV-infected adults attending a Lagos teaching hospital. J Health Popul Nutr. 2010;28(4):318-26.

11. Kampfer P, Matthews H, Glaeser SP, Martin K, Lodders N, Faye I. Elizabethkingia anophelis sp. nov., isolated from the midgut of the mosquito Anopheles gambiae. Int J Syst Evol Microbiol. 2011;61(Pt 11):2670-5.

12. Jiang X, Wang D, Wang Y, Yan H, Shi L, Zhou L. Occurrence of antimicrobial resistance genes sul and dfrA12 in hospital environmental isolates of Elizabethkingia meningoseptica. World J Microbiol Biotechnol. 2012;28(11):3097-102.

13. Tak V, Mathur P, Varghese P, Misra MC. Elizabethkingia meningoseptica: an emerging pathogen causing meningitis in a hospitalized adult trauma patient. Indian J Med Microbiol. 2013;31(3):293-5.

14. Bayrak B, Fincanci M, Binay UD, Cimen C, Ozkantar Unlugunes GU. [Elizabethkingia meningosepticum bacteremia in a patient with Bardet-Biedl syndrome and chronic renal failure]. Mikrobiyol Bul. 2014;48(3):495-500.

15. Shailaja VV, Reddy AK, Alimelu M, Sadanand LN. Neonatal Meningitis by Multidrug Resistant Elizabethkingia meningosepticum Identified by 16S Ribosomal RNA Gene Sequencing. Int J Pediatr. 2014;2014:918907.

16. Arega B, Wolde-Amanuel Y, Adane K, Belay E, Abubeker A, Asrat D. Rare bacterial isolates causing bloodstream infections in Ethiopian patients with cancer. Infect Agent Cancer. 2017;12(1):40.

17. Sedigh Ebrahim-Saraie H, Heidari H, Khashei R, Nabavizadeh SH. A rare case of complicated pericardial effusion with Elizabethkingia meningoseptica from Iran. Cell Mol Biol (Noisy-le-grand). 2018;64(3):53-5.

18. Johnson WL, Ramachandran A, Torres NJ, Nicholson AC, Whitney AM, Bell M, et al. The draft genomes of Elizabethkingia anophelis of equine origin are genetically similar to three isolates from human clinical specimens. PLoS ONE. 2018;13(7):e0200731.

19. Chang T-YC, Hsing-Yu Chou, Yu-Ching Cheng, Yun-Hsiang Sun, Jun-Ren. In vitro activities of imipenem, vancomycin, and rifampicin against clinical <i>Elizabethkingia</i> species producing BlaB and GOB metallo-beta-lactamases. European Journal of Clinical Microbiology & Infectious Diseases. 2019;38(11):2045-52.

20. Joshi P, Shah B, Joshi V, Kumar A, Singhal T. Treatment of Elizabethkingia meningoseptica Neonatal Meningitis with Combination Systemic and Intraventricular Therapy. Indian J Pediatr. 2019;86(4):379-81.

21. Naidenov B, Lim A, Willyerd K, Torres NJ, Johnson WL, Hwang HJ, et al. Pan-Genomic and Polymorphic Driven Prediction of Antibiotic Resistance in Elizabethkingia. Front Microbiol. 2019;10:1446.

22. Barnawi AI, Kordy FN, Almuwallad OK, Kassarah KA. Early neonatal sepsis and meningitis caused by Elizabethkingia meningoseptica in Saudi Arabia. Saudi Med J. 2020;41(7):753-6.

23. Burnard D, Gore L, Henderson A, Ranasinghe A, Bergh H, Cottrell K, et al. Comparative Genomics and Antimicrobial Resistance Profiling of Elizabethkingia Isolates Reveal Nosocomial Transmission and In Vitro Susceptibility to Fluoroquinolones, Tetracyclines, and Trimethoprim-Sulfamethoxazole. J Clin Microbiol. 2020;58(9).

24. Hu R, Zhang Q, Gu Z. Whole-genome analysis of the potentially zoonotic Elizabethkingia miricola FL160902 with two new chromosomal MBL gene variants. J Antimicrob Chemother. 2020;75(3):526-30.

25. Johnston GCA, Lumsden JM. Antimicrobial susceptibility of bacterial isolates from 33 thoroughbred horses with arytenoid chondropathy (2005-2019). Vet Surg. 2020;49(7):1283-91.

26. Seong H, Kim JH, Kim JH, Lee WJ, Ahn JY, M DN, et al. Risk Factors for Mortality in Patients with Elizabethkingia Infection and the Clinical Impact of the Antimicrobial Susceptibility Patterns of Elizabethkingia Species. J Clin Med. 2020;9(5).

27. Wang L, Chen H, Liu W, Yang L, Xu Z, Chen D. Resistome and Genome Analysis of an Extensively Drug-Resistant Klebsiella michiganensis KMIB106: Characterization of a Novel KPC Plasmid pB106-1 and a Novel Cointegrate Plasmid pB106-IMP Harboring bla(IMP-4) and bla(SHV-12). Antibiotics (Basel). 2023;12(9).

28. Costa ARd, Chideroli RT, Chicoski LM, Abreu DCd, Favero LM, Ferrari NA, et al. Frequency of pathogens in routine bacteriological diagnosis in fish and their antimicrobial resistance. Semina: Ciências Agrárias. 2021;42(6):3259-72.

29. Kuo SC, Tan MC, Huang WC, Wu HC, Chen FJ, Liao YC, et al. Susceptibility of Elizabethkingia spp. to commonly tested and novel antibiotics and concordance between broth microdilution and automated testing methods. J Antimicrob Chemother. 2021;76(3):653-8.

30. Xu L, Peng B, He Y, Cui Y, Hu Q, Wu Y, et al. Isolation of Elizabethkingia anophelis From COVID-19 Swab Kits. Front Microbiol. 2021;12:799150.

31. Hashmi AW, Ahmad M, Israr MM, Fajar IE, Adnan F. Multi-Drug-Resistant Elizabethkingia meningoseptica: A Rare Cause of Late-Onset Sepsis in a Preterm Neonate. Cureus. 2023;15(1):e34361.

32. Li S, Wang X, Lu Y, Wang J, Yu D, Zhou Z, et al. Co-infections of Klebsiella pneumoniae and Elizabethkingia miricola in black-spotted frogs (Pelophylax nigromaculatus). Microb Pathog. 2023;180:106150.

33. Sarathi S, Behera B, Mahapatra A, Mohapatra S, Jena J, Nayak S. Microbiological Characterization and Clinical Facets of Elizabethkingia Bloodstream Infections in a Tertiary Care Hospital of Eastern India. Infect Drug Resist. 2023;16:3257-67.

34. Wei D, Cheng Y, Xiao S, Liao W, Yu Q, Han S, et al. Natural occurrences and characterization of Elizabethkingia miricola infection in cultured bullfrogs (Rana catesbeiana). Front Cell Infect Microbiol. 2023;13:1094050.
